# 基于二代测序分析伴RAS突变初诊正常核型急性髓系白血病患者的临床特征及预后

**DOI:** 10.3760/cma.j.issn.0253-2727.2023.09.010

**Published:** 2023-09

**Authors:** 弘正 梁, 艳萍 马, 林花 杨, 巧花 郭, 世芳 王, 参 李

**Affiliations:** 山西医科大学第二医院血液科，太原 030001 Institute of Hematology, The Second Hospital of Shanxi Medical University, Taiyuan 030001, China

急性髓系白血病（AML）是一类骨髓原始幼稚细胞恶性增殖性疾病，白血病干细胞由于增殖失控、分化障碍和细胞凋亡受阻等原因，产生大量未成熟的异常髓系细胞堆积在骨髓和外周血中，导致骨髓正常造血功能受损和功能衰竭[1]–[2]。研究发现AML的发生与染色体异常、基因突变以及基因表达异常有关[3]。目前正常核型被认为是AML患者预后中等的一个标志，但由于患者往往可伴随不同突变且具有不同的克隆，而导致预后具有极大的异质性。RAS在AML中突变率约为30％，可参与多种肿瘤的形成与发展，与预后不良相关[4]–[6]。目前关于RAS突变正常核型AML（CN-AML）患者的临床特征、克隆特点及RAS突变对预后的影响仍有争议。本研究分析初诊CN-AML中RAS突变患者的临床特点及RAS突变对预后的影响。

## 病例与方法

1. 一般资料：回顾性分析2016年12月至2021年12月在山西医科大学第二医院中心诊治的148例初诊CN-AML患者，所有入组患者均完成骨髓细胞形态学、免疫分型、染色体核型、融合基因（MICM）分型检测，收集患者初诊时的年龄、性别、血常规、LDH、骨髓原始细胞比例、流式免疫分析、染色体核型、危险度分层及二代测序等资料。

2. 治疗方案：参照文献[1]，所有患者接受蒽环类药物联合阿糖胞苷标准诱导化疗方案（"3+7"方案），诱导化疗达完全缓解（CR）后给予大剂量阿糖胞苷（3 g/m^2^每12 h 1次，共6次）3～4个疗程。61例患者在化疗期间联合地西他滨或阿扎胞苷进行巩固强化。20例患者接受异基因造血干细胞移植治疗。

3. 靶向二代测序：应用二代测序方法对患者ASXL1、BOCR、BOCRL1、CALR、CBL、CSF3R、DNMT3A、ETV6、EZH2、FLT3、GATA2、IDH1/2、JAK2、KIT、KRAS、MLL、MPL、NOTHCH2、NPM1、NRAS、PDGFRA、PHF6、PIGA、RUNX1、SETBP1、SH2B3、SF3B1、SRSF2、TET2、TP53、U2AF1、WT1、ZRSR2等34种常见髓系相关基因突变进行检测。变异负荷（VAF）<5％不纳入分析。

4. 克隆等级分析：参照Esther Onecha团队在评估AML中突变的主亚克隆判定方法以及根据二代测序结果推断AML中ASXL1、PTPN1等突变的克隆等级分类方法[2],[7]–[8]，同时参考肖志坚教授团队[9]判定骨髓增殖性肿瘤中RAS突变主亚克隆的方式。我们以RAS突变的VAF≥10％其共突变基因，则认为RAS突变为主克隆；RAS突变的VAF<10％其共突变基因，则认为RAS突变为亚克隆。

5. 随访及相关定义：所有患者采用查阅病历或电话的方式进行随访。随访截至2021年12月1日。CR：①临床无贫血、出血、感染及白血病细胞浸润表现；②HGB>90 g/L，白细胞计数正常或减低，分类无幼稚细胞，PLT>100×10^9^/L；③原始细胞加早幼阶段细胞（或幼稚细胞）<5％，红细胞系统及巨核细胞系统正常。复发：血液或骨髓中的原始细胞再次>5％或在达CR后的任何髓外部位复发。总生存（OS）期：患者开始治疗至死亡或末次随访的时间。无复发生存（RFS）期：患者化疗达到CR后至疾病复发或因任何原因导致死亡的时间[1]。

6. 统计学处理：采用SPSS 25.0软件进行分析，连续变量比较采用独立样本的Mann-Whtney *U*检验（不符合正态分布），分类变量比较采用Fisher精确概率法。生存分析采用Kaplan-Meier法，组间比较采用Log-rank检验，采用Cox比例风险模型进行多因素分析。*P*<0.05为差异有统计学意义。

## 结果

1. 初诊CN-AML中RAS基因突变情况：根据二代测序结果，148例初诊CN-AML患者中，22例（14.9％）检出27个RAS突变，均为错义突变，且所有RAS突变位点均为热点突变。其中单纯NRAS突变16例，单纯KRAS突变3例，同时伴有NRAS与KRAS突变3例。RAS突变类型：NRAS突变以G12D（7/21）、G13D（6/21）位点为主，KRAS突变为G13D（2/6）与G12D（4/6）。RAS突变的中位VAF为22.7％（12.1％，38.4％），克隆等级分析显示亚克隆突变占54％，主克隆突变占46％（表1）。

**表1:** 22例伴RAS基因突变初诊正常核型急性髓系白血病（CN-AML）患者信息及RAS突变特征

编号	性别	年龄	危险度分层	基因	DNA序列改变	蛋白质序列改变	突变类型	变异负荷（VAF）	克隆等级
1	男	71	预后良好	NRAS	G35A	G12D	错义突变	38.4%	主克隆
2	女	21	预后良好	NRAS	G35T	G12V	错义突变	42.8%	主克隆
3	男	72	预后中等	NRAS	G38A	G13D	错义突变	16.5%	亚克隆
4	男	49	预后良好	KRAS	G35A	G12D	错义突变	18.0%	亚克隆
5	男	64	预后中等	NRAS	G38A	G13D	错义突变	6.4%	亚克隆
6	男	72	预后良好	KRAS	G38A	G13D	错义突变	12.1%	亚克隆
7	男	21	预后不良	NRAS	G35A	G12D	错义突变	8.9%	亚克隆
8	男	21	预后不良	NRAS	G35A	G12D	错义突变	15.4%	亚克隆
				NRAS	A182G	Q61R	错义突变	7.8%	亚克隆
				KRAS	G38A	G13D	错义突变	9.0%	亚克隆
9	女	27	预后不良	KRAS	G35A	G12D	错义突变	29.3%	亚克隆
10	男	29	预后中等	NRAS	A183T	Q61H	错义突变	12.3%	亚克隆
				NRAS	C181A	Q61K	错义突变	12.0%	亚克隆
11	男	53	预后良好	NRAS	G35A	G12D	错义突变	35.4%	亚克隆
12	男	63	预后中等	NRAS	G34T	G12C	错义突变	28.3%	主克隆
				KRAS	G35A	G12D	错义突变	15.1%	亚克隆
13	男	36	预后良好	NRAS	G34T	G12C	错义突变	46.0%	主克隆
14	男	29	预后中等	NRAS	G38A	G13D	错义突变	41.6%	主克隆
15	男	61	预后良好	NRAS	G35A	G12D	错义突变	22.7%	主克隆
16	男	45	预后良好	NRAS	G35C	G12A	错义突变	8.0%	亚克隆
17	男	64	预后良好	NRAS	G38A	G13D	错义突变	44.4%	主克隆
18	男	27	预后中等	NRAS	G35A	G12D	错义突变	25.0%	亚克隆
19	男	29	预后中等	NRAS	G35A	G12D	错义突变	13.1%	主克隆
20	男	58	预后中等	NRAS	G38A	G13D	错义突变	44.4%	主克隆
21	男	19	预后中等	NRAS	G38A	G13D	错义突变	44.4%	主克隆
				KRAS	G35A	G12D	错义突变	32.0%	亚克隆
22	男	43	预后良好	NRAS	G35A	G12D	错义突变	30.0%	亚克隆

2. RAS突变的CN-AML患者临床特征及伴随突变特点：RAS突变患者中男女各11例，中位年龄44（27，63）岁。实验室分析显示：RAS突变组中位WBC为28.37（10.44，262.69）×10^9^/L，高于RAS未突变组（*P*＝0.014），中位单核细胞计数6.55（2.03，27.66）×10^9^/L，也明显高于RAS未突变组（*P*＝0.003）。同时发现RAS突变组中位LDH（*P*＝0.004）以及α-羟丁酸脱氢酶（HBDH）（*P*＝0.012）亦均明显高于RAS未突变组。为进一步明确RAS突变患者骨髓原始细胞特征，对所有初诊CN-AML患者骨髓流式分析显示：RAS突变组骨髓原始细胞CD34表达阳性率显著低于RAS未突变组（36％对73％，Fisher，*P*＝0.001），其余差异均无统计学意义（表2）。

**表2:** 伴RAS基因突变的初诊正常核型急性髓系白血病（CN-AML）患者临床特征

指标	总体	RAS 突变组（22例）	RAS 未突变组（126例）	统计量	*P*值
年龄［例（％）］				0.054	0.816
≥60岁	44（30）	7（32）	37（29）		
<60岁	104（70）	15（68）	89（71）		
性别［例（％）］				0.043	0.837
男	77（52）	11（50）	66（52）		
女	71（48）	11（50）	60（28）		
实验室检查[*M*（*Q*_1_, *Q*_3_）]				
RBC（×10^12^/L）	2.39（1.91, 3.11）	2.7（2.14, 3.43）	2.31（1.87, 3.05）	−1.195	0.232
WBC（×10^9^/L）	11.48（3.08, 38.81）	28.37（10.44, 262.69）	8.71（2.71, 36.07）	−2.451	0.014
PLT（×10^9^/L）	39（22, 76）	40.5（24, 79）	39（22, 73）	−0.462	0.664
HGB（g/L）	81（69, 101）	89（76, 106）	78（68, 100）	−1.414	0.157
单核细胞计数（×10^9^/L）	1.71（0.27, 11.47）	6.55（2.03, 27.66）	1.23（0.22, 7.59）	−2.991	0.003
骨髓原始细胞比例（％）	58（35, 76）	50（39, 66）	62（33, 78）	−0.953	0.330
LDH（U/ml）	389（242, 589）	550（381, 893）	361（233, 547）	−2.892	0.004
HBDH（U/ml）	311（194, 483）	413（291, 686）	275（180, 446）	−2.526	0.012
危险度分层［例（％）］				8.828	0.012
预后良好组	32（22）	10（45）	22（17）		
预后中等组	80（54）	9（41）	71（56）		
预后不良组	36（24）	3（14）	33（27）		
接受allo-HSCT	20（14）	1（5）	19（15）	0.991	0.319

**注** HBDH：α-羟丁酸脱氢酶；allo-HSCT：异基因造血干细胞移植

我们进一步分析伴RAS突变是否存在易伴随或互斥的突变基因，根据二代测序结果分析显示：RAS突变的CN-AML患者中，伴随NPM1、DNMT3A、TET2以及CEBPA突变频率分别为41％、32％、27％和18％。与RAS未突变组相比，RAS突变的患者更易伴随NPM1与DNMT3A突变。

3. RAS突变克隆演进：在22例RAS突变患者中，RAS突变主克隆10例，亚克隆12例。其中2例RAS突变患者进行多次二代测序，结果显示，RAS突变无论是主克隆还是亚克隆，在化疗达CR时除胚系突变外所有突变变异负荷均明显降低或未检出。而在复发时，该克隆同时可能伴随有额外突变（如CEBPA等）且RAS及其伴随突变的变异负荷也明显增加（图1）。

**图1:**
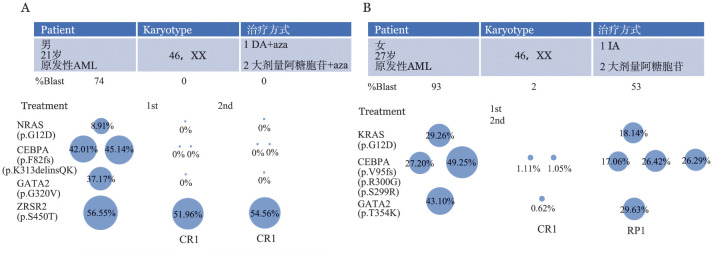
2例伴RAS基因突变的初诊正常核型急性髓系白血病（CN-AML）患者RAS突变克隆演进 **A** NRAS突变（亚克隆）；**B** KRAS突变（主克隆）

4. RAS突变的CN-AML患者预后分析：RAS突变组与RAS未突变组患者完全缓解（CR）率分别为95％、82％，差异无统计学意义（Fisher，*P*＝0.371）；复发率分别为45％对26％，差异无统计学意义（Fisher，*P*＝0.078）；RAS突变患者OS相较于RAS未突变组差异无统计学意义（*P*＝0.084），但RAS突变组患者RFS时间明显缩短（*P*＝0.029）。由于RAS突变患者易伴随有NPM1、DNMT3A、TET2突变，我们进一步分析伴随突变是否对RAS突变的CN-AML患者的预后产生影响，结果如图2所示：在RAS突变的CN-AML患者中，RAS/NPM1双突变组患者的OS时间相较于RAS突变/NPM1未突变组患者差异无统计学意义（*P*＝0.186），但明显短于RAS未突变组（*P*＝0.008）。RAS/NPM1双突变组患者RFS（*P*＝0.024）明显低于RAS突变/NPM1未突变组与RAS未突变组（*P*<0.001）；同样，RAS突变的CN-AML患者在伴随DNMT3A（*P*＝0.005）或TET2突变（*P*＝0.007）时RFS时间明显缩短。10例主克隆与12例亚克隆两组患者在OS（*P*＝0.281）及RFS（*P*＝0.820）差异均无统计学意义。在148例患者中部分患者在治疗过程中可能联合地西他滨或阿扎胞苷，其中RAS突变且联合地西他滨或阿扎胞苷进行强化巩固12例，相较于标准治疗组，联合地西他滨或阿扎胞苷可明显延长患者OS时间（*P*＝0.003）与RFS时间（*P*＝0.007）。

**图2:**
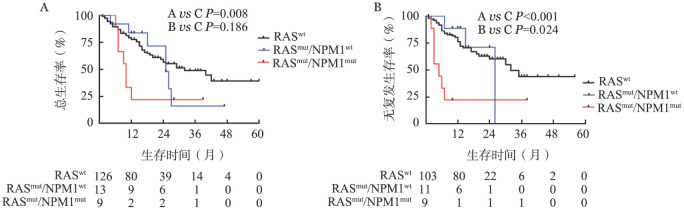
RAS突变伴随NPM1突变对于初诊正常核型急性髓系白血病（CN-AML）患者总生存（A）与无复发生存（B）曲线

5. 多因素分析：基于上述分析发现，RAS突变对于患者RFS有显著影响，为明确RAS突变是否为影响预后的独立影响因素，我们将影响患者预后的因素纳入多因素分析，结果图3所示，RAS突变不是影响CN-AML患者OS（*HR*＝1.315，95％*CI* 0.587～2.943）与RFS（*HR*＝1.403，95％*CI* 0.501～3.929）预后不良的独立影响因素。

**图3:**
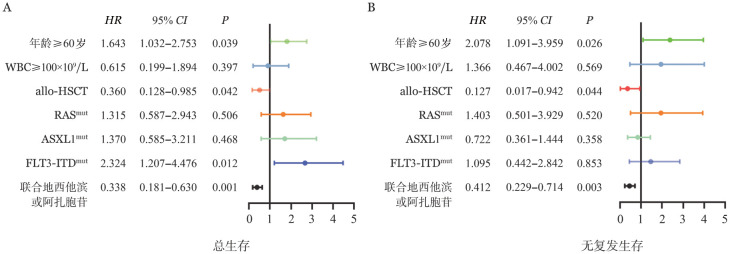
影响初诊正常核型急性髓系白血病（CN-AML）总生存（A）与无复发生存（B）的多因素分析

## 讨论

RAS基因编码的RAS蛋白经翻译后修饰可定位于细胞膜内侧，通过RAS/RAF/MEK和RAS/PI3K/AKT等信号通路传导细胞信号，调控细胞的增殖、分化和凋亡[10]。根据Knudson[11]的2次打击理论，AML发生包括两个遗传事件发生：Ⅰ类突变与Ⅱ类突变，其中I类突变介导增殖信号，Ⅱ类突变损害造血细胞分化，RAS突变属于Ⅰ类突变。虽然RAS基因包括KRAS、NRAS和HRAS三种，但HRAS突变占比小于1％且在血液疾病中检出率低，因此本研究对HRAS不予分析[4],[12]。

目前大部分研究队列中包括异常核型患者中，RAS突变在初诊AML中发生频率为15％～20％，但对于RAS突变在CN-AML中的作用及预后仍不明确[4]–[6],[13]–[14]。本研究中，我们通过对148例初诊CN-AML患者二代测序分析发现，RAS在CN-AML中突变检出率为15％，与AML总体一致。同时，RAS突变位点均以热点突变为主，其中G12D位点突变是主要类型，与国内外研究一致，表明RAS突变相对保守，研究发现，内源性NRAS G12D位点突变过度激活造血干细胞中的ERK1/2从而导致骨髓造血干/祖细胞扩增，并增强其自我更新能力，在抑制RAS或者MEK可明显使患者获益[15]–[16]。RAS突变的中位VAF值较低且以亚克隆为主，Akram等[17]研究亦支持此观点，提示RAS突变常为疾病发生晚阶段的分子事件，RAS通路的调控紊乱使AML细胞获得生长增殖优势，并在AML的发生发展中发挥关键作用。

在本次分析中发现，RAS突变组中位LDH以及HBDH均明显高于RAS未突变组，与国内外报道一致，表明RAS突变对于LDH、HBDH的表达可能存在调控关系，仍需后续进一步研究以证明其可能存在的内在联系。研究表明AML患者中NRAS突变易伴随NPM1突变，Padmakumar等[18]发现其二者共突变频率高，我们的分析结果也发现在CN-AML中有此现象。我们进一步分析NPM1与RAS共突变这个亚群，发现RAS的VAF均低于NPM1，且RAS突变以亚克隆为主，而NPM1突变却以主克隆为主。目前认为NPM1作为AML发生的早期分子事件并为预后良好的分子指标，但本次分析发现NPM1与RAS共突变患者RFS时间明显缩短，对此我们推测NPM1突变可能会利于RAS突变的发生并促进AML的发生发展。目前NPM1与RAS共突变预后不良的原因的机制研究报道较少，且对于RAS突变以亚克隆为主的现象目前也无法解释，NPM1突变是否会诱发RAS突变仍需进一步研究。在对2例患者RAS突变患者克隆演进分析时发现，在初次化疗时反应良好，化疗达CR时除胚系突变外所有突变变异负荷均明显降低或未检出。但在该克隆再次伴随有突变时复发。提示我们在达到CR时还应加强对突变基因的VAF检测，同时可能应使患者尽量达到更深层次的分子水平缓解，减少复发。

虽然目前对于RAS突变在AML中研究较多，但对于RAS突变的主亚克隆对预后的作用研究较少，我们进一步预后分析发现RAS主亚克隆间的预后差异无统计学意义，因本研究病例数较少，此观点我们仍需收集数据进一步分析，同时也期待更多研究团队的报道RAS不同克隆与预后的关系。我们的结果还表明RAS突变不是影响CN-AML患者独立的不良预后因素，与RAS在AML中不是独立的不良预后因素观点一致[17]。

本研究为单中心回顾性分析，限于病例数目，结论仍需多中心大样本前瞻性研究加以证实。
